# *Bifidobacterium breve* CCFM1025 Improves Sleep Quality via Regulating the Activity of the HPA Axis: A Randomized Clinical Trial

**DOI:** 10.3390/nu15214700

**Published:** 2023-11-06

**Authors:** Yuming Lan, Junjie Lu, Guohong Qiao, Xuhua Mao, Jianxin Zhao, Gang Wang, Peijun Tian, Wei Chen

**Affiliations:** 1 State Key Laboratory of Food Science and Resources, Jiangnan University, Wuxi 214122, China; 6210113187@stu.jiangnan.edu.cn (Y.L.); zhaojianxin@jiangnan.edu.cn (J.Z.); wanggang@jiangnan.edu.cn (G.W.); chenwei66@jiangnan.edu.cn (W.C.); 2 School of Food Science and Technology, Jiangnan University, Wuxi 214122, China; 3 National Engineering Research Center for Functional Food, Jiangnan University, Wuxi 214122, China; 4 Department of Critical Care Medicine, Yixing People’s Hospital Affiliated Jiangsu University, Yixing 214200, China; staff806@yxph.com (J.L.); staff1291@yxph.com (X.M.); 5 Department of Clinical Laboratory, Yixing People’s Hospital Affiliated Jiangsu University, Yixing 214200, China; staff1000@yxph.com; 6 (Yangzhou) Institute of Food Biotechnology, Jiangnan University, Yangzhou 225004, China

**Keywords:** probiotics, sleep, insomnia, stress, HPA axis, daidzein

## Abstract

Psychobiotics, a newly identified category of probiotics primarily targeting the gut–brain axis, exhibit tremendous potential in improving sleep quality. In this study, the clinical trial was registered in advance (identifier: NO. ChiCTR2300067806). Forty participants who were diagnosed with stress-induced insomnia were chosen and randomly divided into two groups: one received CCFM1025 at a dose of 5 × 10^9^ CFU (*n* = 20), while the other was administered a placebo (*n* = 20), over a period of four weeks. The results revealed that compared to the placebo group (pre: M = 10.10, SD = 2.292; post: M = 8.650, SD = 2.793; pre vs. post: F (1, 38) = 15.41, *p* = 0.4316), the CCFM1025-treated group exhibited a significant decrease in Pittsburgh Sleep Quality Index (PSQI) scores from baseline (pre: M = 11.60, SD = 3.169; post: M = 7.750, SD = 3.697, F (1, 38) = 15.41, *p* = 0.0007). Furthermore, the administration of CCFM1025 was associated with a more pronounced reduction in stress marker concentrations. This effect could potentially be linked to changes in serum metabolites induced by the probiotic treatment, notably daidzein. In conclusion, *B. breve* CCFM1025 demonstrates promise as a psychobiotic strain for enhancing sleep quality.

## 1. Introduction

Natural sleep, constituting about one-third of an individual’s lifespan, plays a vital role in various aspects of human health, including neurodevelopment, learning, memory, emotional regulation, and cardiovascular and metabolic functions, as well as the clearance of cytotoxins [1]. However, numerous factors, both physiological and environmental, can disrupt sleep patterns, resulting in insufficient and poor-quality sleep. These factors encompass conditions like insomnia, obstructive sleep apnea, circadian rhythm disorders, and even occupational stress [2]. The consequences of chronic sleep deprivation are far-reaching, contributing to inflammation, metabolic dysfunction, cognitive impairment, and an increased risk of mental illnesses [3,4]. In the long term, inadequate sleep has been associated with the development of serious health conditions such as diabetes, hypertension, cardiovascular disease, stroke, coronary heart disease, obesity, and depression [5]. Insomnia disorder (ID) is particularly noteworthy, being the most prevalent sleep disorder and ranking as the second most common neuropsychiatric disorder [6]. Recent research conducted by Li et al. [7] showed the extensive prevalence of insomnia symptoms within the Chinese population, with rates as high as 39.1%. Numerous prior studies have also highlighted the close relationship between insufficient sleep and elevated levels of job-related stress [8].

The gut–brain axis has garnered increasing attention in the realms of neurology, psychiatry, neurodevelopment, and neurodegenerative diseases due to its profound biological and physiological implications [9]. Gut microbes mediate brain function and host behaviors either directly or indirectly, including immune pathways (immune cells and cytokines), endocrine pathways (HPA axis), and neural pathways (neurotransmitters, neuroactive metabolites, vagus nerve, enteric nervous system, and spinal nerve) [10]. In fact, several studies have indicated that treatments using psychobiotics may decrease stress and improve sleep quality. For example, *Lactobacillus gasseri* CP2305 can alleviate stress and improve sleep in young adults by regulating gut microbiota composition and reducing salivary cortisol and other stress indicators [11,12]. Using miniature polysomnography (PSG), Ho et al. discovered that a four-week intervention with probiotic *Lactobacillus plantarum* PS128 could affect sleep architecture and improve the quality of deep sleep [13]. These findings revealed the therapeutic potential of probiotics in stress management and the alleviation of stress-related symptoms, such as insomnia.

*Bifidobacterium breve* CCFM1025, as a potential psychoprobiotic strain, has a positive regulatory effect on depression, Alzheimer’s disease, and other mental diseases [14,15]. The underlying mechanisms may involve the alleviation of HPA axis hyperactivity, suppression of the inflammatory response, modulation of the serotonergic system, and alteration of the gut microbiota composition [16]. In addition, CCFM1025 was found to colonize in the human gut [17]. In our prior research, we observed that *Bifidobacterium breve* CCFM1025 effectively alleviated anxiety and improved sleep quality in participants with depression [18]. To gain deeper insights, animal studies were conducted, showing that CCFM1025 influenced the production of key metabolites involved in regulating sleep function through the modulation of gut microbiota [19]. This modulation, in turn, mitigated circadian rhythm disturbances induced by sleep deprivation in mice. Building on these significant findings, we initiated a clinical trial to investigate the impact of CCFM1025 intervention on sleep quality in individuals suffering from insomnia. In contrast to previous approaches, our study specifically focused on evaluating the probiotic’s effect on participants’ hyperactivity of the HPA axis.

## 2. Materials and Methods

### 2.1. Ethical Approval

This trial was approved by Yixing People’s Hospital (Yixing, China, Approval Code: Lunshen2022Ke188). The registration number of the Chinese Clinical Trial Registry was ChiCTR2300067806.

### 2.2. Sample Size Determination

The software G*Power3.1 was used to analyze prior power. We estimated the effect size from a published study that improved sleep quality by comparing the mean difference of the Pittsburgh Sleep Quality Index (PSQI) between the control groups and treated groups [20]. In order to attain a statistical power of 0.8 for a t-test analysis, it is necessary to have groups consisting of a minimum of eighteen participants, resulting in an effect size of d = 0.847 at a significance level of α = 0.05 (See Supplemental Figure S1 for details). We recruited 20 volunteers per group.

### 2.3. Study Design

**Figure 1:**
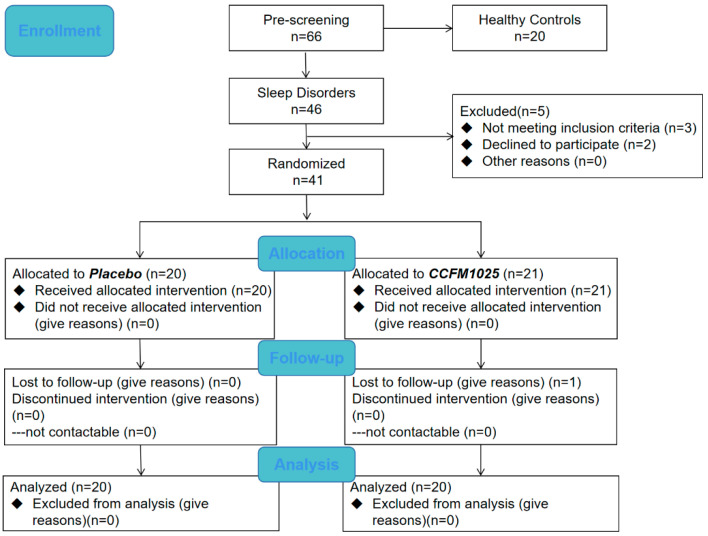
Flow chart.

This study was a placebo-controlled, randomized controlled trial (RCT). The CONSORT flow diagram is shown in Figure 1. Participants with an age range of 18–65 years were divided into healthy control groups and sleep disorder groups using the PSQI score before enrollment. The sleep disorder group was then divided into placebo and CCFM1025-treated groups. Participants were blinded and received the intervention product independently, administered by an authorized investigator, following a 1:1 allocation ratio determined by a randomization code [21]. Samples of the healthy control group were only taken at baseline, whereas samples of the sleep disorder group were taken at baseline and after intervention. All of the samples were collected at the hospital under the guidance of another authorized investigator. With a PSQI score > 7, participants with the following symptoms such as difficulty falling asleep and waking up early were diagnosed with insomnia. Participants who take sleep aids, work night shifts, have an irregular lifestyle, or have a history of alcohol dependence, pregnant or lactating women, and men or women who are preparing for pregnancy (childbearing) were excluded. As a result, 60 participants completed the trial (health control group, *n* = 20, female ratio 50%; placebo, *n* = 20, female ratio 70%; CCFM1025, *n* = 20, female ratio 60%). The demographic characteristics are presented in Table 1. (The demographic information of all participants including the healthy control group and the sleep disorder group is shown in Supplemental Table S1).

The probiotic treatment group received a daily sachet consisting of *Bifidobacterium breve* CCFM1025 in a dose of 10^9^ CFU per day and maltodextrin, while the placebo group received an identical-appearing sachet containing only maltodextrin for four weeks. (Specific experimental schedule is shown in Supplemental Figure S2). The products were produced by Shisheng Yisheng, Co., Ltd. (Yangzhou, China). Participants were asked to avoid supplements and food containing probiotics during their participation in the study, with no other dietary restrictions. Enrollment and sample collection began on 1 January 2023 and ended on 31 March 2023. Sample collection and data analysis were completed in the laboratory in June 2023.

### 2.4. Sleep Disorder Measurements (Major Outcome Measures)

PSQI was used to quantify sleep quality as a major experimental outcome [22]. Higher total PSQI scores indicate poorer sleep quality. A PSQI score greater than 7 was used as the cut-off point for whether a participant had a sleep disorder. We recruited participants with a total PSQI score > 7. Participants were scored during routine examinations. In addition, the Athens Insomnia Scale (AIS) was used as an auxiliary judgment for sleep quality.

### 2.5. Salivary Cortisol Measurements

Salivary cortisol level was measured at the start and after a 4-week period to assess the HPA axis’s activity in relation to chronic stress [23]. Since the secretion of cortisol peaks around 9 a.m. [24], salivary samples were collected at this time. Saliva samples from all participants were collected using a commercial saliva tube (Shanghai Zhizhi Biotechnology Co., Ltd., Shanghai, China) and centrifuged according to the instructions to remove particulate matter. The quantification of free cortisol was performed using a saliva cortisol ELISA kit (Shanghai Coibo Bio Technology Co., Ltd., Shanghai, China), according to the manufacturer’s instructions.

### 2.6. Plasma Measurements

After saliva collection, 4 mL of fasting peripheral venous blood was taken from participants. Anticoagulant was added into the blood collection tube, and plasma was separated by 3000 r/min and stored in a low temperature freezer (−80 °C). The Cortisol CLIA Microparticles (Autobio Diagnostics Co., Ltd., Zhengzhou, China) and the ACTH CLIA Microparticles (Autobio Diagnostics Co., Ltd.) were used to detect the levels of cortisol and adrenocorticotropin (ACTH) in plasma.

### 2.7. Serum Measurements

The procedure for serum metabolite extraction was described extensively in the study conducted by Yuan et al. [25]. To conduct the metabolomic analysis, we utilized the online software MetaboAnalyst 5.0. For further details, the official website of the software is http://www.metaboanalyst.ca/ (accessed on 15 July 2023). The parameters for the Ultraperformance Liquid Chromatography–Mass Spectrometry (UPLC-MS) analysis are provided in Supplemental S1.1.

### 2.8. Statistical Analysis

Before the commencement of statistical analysis, all clinical data underwent an initial normality test using the Shapiro–Wilk test. IBM SPSS 22.0 was employed for conducting the statistical analyses. To compare the two groups, including the baseline clinical data between the groups, as well as the score and concentration changes between the CCFM1025 and placebo groups, an unpaired Student’s *t*-test was utilized. Cohen’s d was used to assess the effect size. For the analysis of the alterations in scale scores and substance concentrations before and after the intervention in the two groups, a two-way analysis of variance (ANOVA) was conducted, followed by Tukey’s multiple comparisons. The correlation between the compounds was examined using the Pearson correlation coefficient. Statistical significance was determined by considering a *p*-value of less than 0.05 for all comparisons. Graphs were generated using Prism 8.0.

## 3. Results

### 3.1. Clinical Grouping and Validation of Insomnia

**Figure 2:**
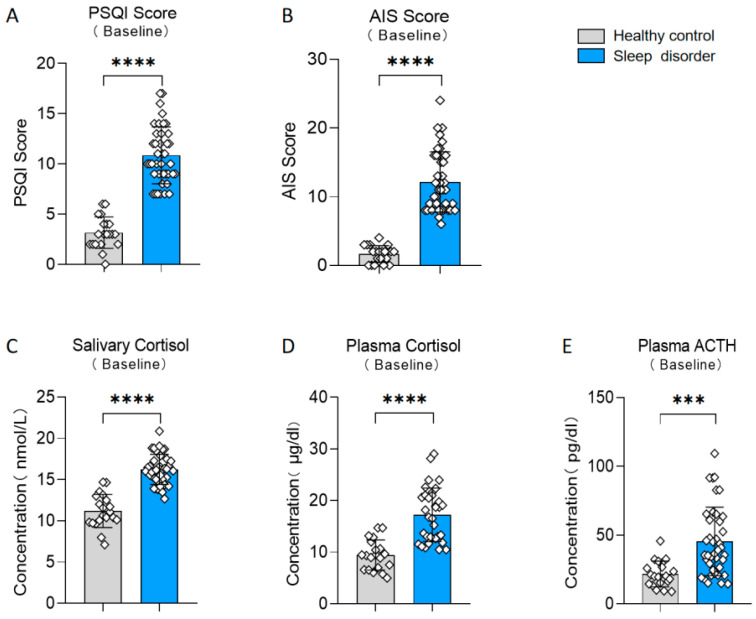
Differences between the sleep disorder and healthy control group. (**A**) Baseline PSQI score. (**B**) Baseline AIS score. (**C**) Baseline salivary cortisol. (**D**) Baseline plasma cortisol. (**E**) Baseline plasma ACTH. *** *p* < 0.001, **** *p* < 0.0001 in the unpaired *t*-tests.

Participants were divided into two groups, a sleep disorder group and a healthy control group, based on their scores on the PSQI (t (58) = 11.29, *p* < 0.0001, d = 2.813; see Figure 2A). Additionally, there existed notable disparities in the results of the AIS between the two groups (t (58) = 10.42, *p* < 0.0001, d = 2.598; see Figure 2B). We also measured various physiological indicators in both groups. The sleep disturbance group exhibited a significantly elevated salivary cortisol level in comparison to the healthy control group (t (58) = 9.774, *p* < 0.0001, d = 2.631; see Figure 2C). Furthermore, the plasma cortisol and ACTH levels in the sleep disorder group were significantly elevated compared to those in the healthy control group (plasma cortisol: t (58) = 6.175, *p* < 0.0001, d = 1.553; see Figure 2D; plasma ACTH: t (58) = 4.093, *p* = 0.0001, d = 1.020; see Figure 2E). These results align with previous studies [26,27], confirming the reliability of our insomnia assessment.

### 3.2. CCFM1025 Intervention Enhanced the Sleep Quality of Insomnia Patients

**Figure 3:**
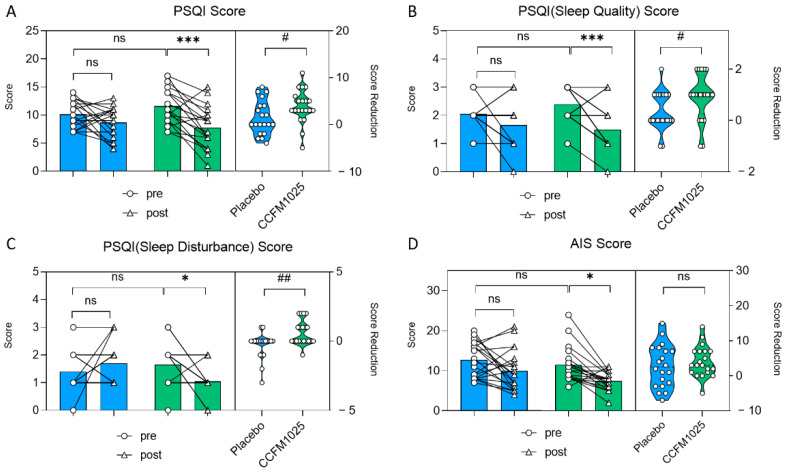
*B. breve* CCFM1025′s effect on sleep improvement. (**A**) Alteration in the score of PSQI. (**B**) The change in rating scores of the PSQI(sleep quality). (**C**) The change in rating scores of the PSQI (sleep disturbance). (**D**) The change in rating scores of AIS. * *p* < 0.05, *** *p* < 0.001 in Tukey’s multiple comparisons test post two-way ANOVA. # *p* < 0.05, ## *p* < 0.01 in the unpaired *t*-tests. ns: not statistically significant.

In the CCFM1025-treated group, the participants’ PSQI score exhibited a notable drop in comparison to the initial baseline measurement (pre vs. post: F (1, 38) = 15.41, *p* = 0.0007; Figure 3A). However, there was no statistical significance in the placebo group (pre vs. post: F (1, 38) = 15.41, *p* = 0.4316; Figure 3A). Moreover, CCFM1025 led to a more significant reduction in the participants’ PSQI score compared to the placebo group (placebo vs. CCFM1025: t (38) = 2.106, *p* = 0.0419, d = 0.666; Figure 3A).

CCFM1025 also improved sleep quality by enhancing participants’ subjective sleep quality and reducing sleep disturbance (sleep quality score: pre vs. post: F (1, 38) =18.19, *p* = 0.004, Figure 3B; sleep disturbance score: pre vs. post: F (1, 38) = 0.937, *p* = 0.038, Figure 3C). Conversely, there was no significant difference in the placebo group (sleep quality score: pre vs. post: F (1, 38) = 18.19, *p* = 0.2557, Figure 3B; sleep disturbance score: pre vs. post: F (1, 38) = 0.937, *p* = 0.5226, Figure 3C).

In the AIS table, AIS scores significantly decreased before and after CCFM1025 intervention, while there was no significant difference in the placebo group before and after intervention (pre-placebo vs. post-placebo: F (1, 38) = 13.46, *p* = 0.1558; pre-CCFM1025 vs. post-CCFM1025: F (1, 38) = 13.46, *p* = 0.0155; Figure 3D).

### 3.3. Effects of CCFM1025 Intervention on Stress Markers in Insomnia Patients

**Figure 4:**
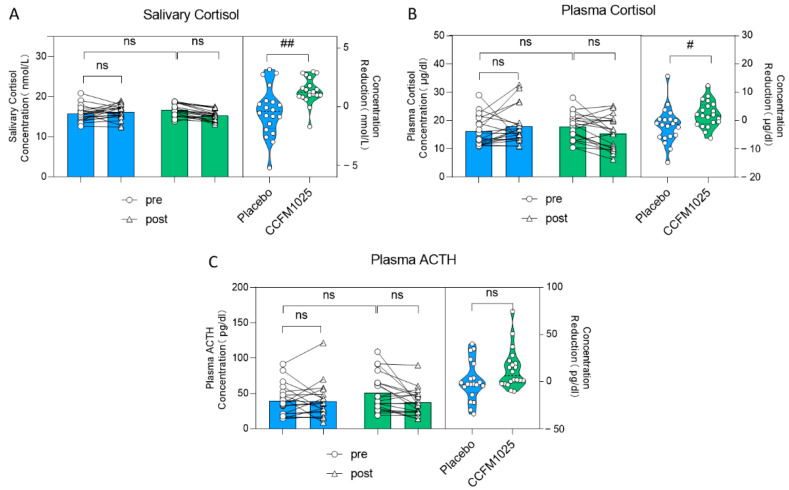
*B. breve* CCFM1025′s effect on stress markers. (**A**) Alteration in the concentration of salivary cortisol. (**B**) The rating concentration change in the plasma cortisol. (**C**) The rating concentration change in the plasma ACTH. # *p* < 0.05, ## *p* < 0.01 in the unpaired *t*-tests. ns: not statistically significant.

Further assessments were conducted by measuring salivary and plasma stress markers. There was no statistically significant difference seen in the level of salivary cortisol. (pre-placebo vs. post-placebo: F (1, 38) = 1.67, *p* = 0.8768; pre-CCFM1025 vs. post-CCFM1025: F (1, 38) = 1.67, *p* = 0.0564; Figure 4A). However, it is noteworthy that the reduction in salivary cortisol concentration between the two groups showed statistical significance. Specifically, compared to the placebo group, CCFM1025 led to a more significant reduction in saliva cortisol concentration (placebo vs. CCFM1025: t (38) = 3.331, *p* = 0.0019, d = 2.764; Figure 4A).

Similar findings were observed in plasma cortisol levels (pre-placebo vs. post-placebo: F (1, 38) = 0.088, *p* = 0.7626; pre-CCFM1025 vs. post-CCFM1025: F (1, 38) = 0.088, *p* = 0.5058; concentration reduction: placebo vs. CCFM1025: t (38) = 2.237, *p* = 0.0312, d = 0.707; Figure 4B). However, it is important to note that after both CCFM1025 intervention and placebo intervention, there were no statistically significant differences in the changes in plasma ACTH levels (pre-placebo vs. post-placebo: F (1, 38) = 1.66, *p* = 0.9997; pre-CCFM1025 vs. post-CCFM1025: F (1, 38) = 1.66, *p* = 0.3141; Figure 4C), and there were no statistically significant differences in the reduction in plasma ACTH content (placebo vs. CCFM1025: t (38) = 1.935, *p* = 0.0605, d = 0.6616; Figure 4C).

### 3.4. Metabolomic Analysis Reveals Daidzein as a Biomarker in CCFM1025 Intervention

**Figure 5:**
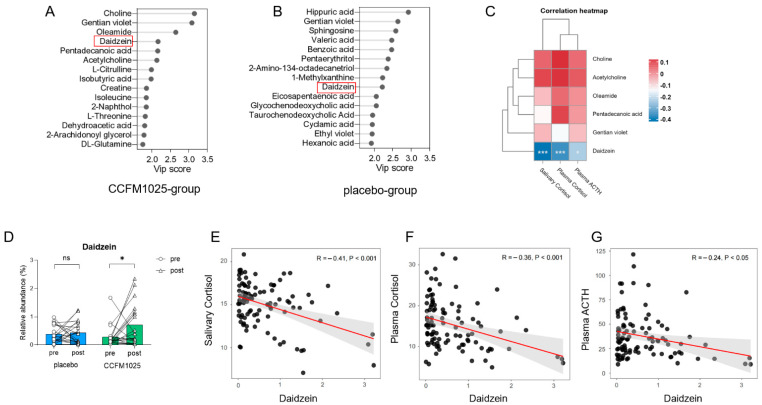
*B. breve* CCFM1025′s effect on serum metabolites. (**A**) Biomarkers of serum metabolites in the CCFM1025 intervention group. (**B**) Biomarkers of serum metabolites in the placebo intervention group. (**C**) Correlation analysis between biomarkers and stress markers. (**D**) The relative abundance changes in the serum daidzein. (**E**) The correlation between salivary cortisol and daidzein. (**F**) The correlation between plasma cortisol and daidzein. (**G**) The correlation between plasma ACTH and daidzein. * *p* < 0.05 in the unpaired *t*-tests. ns: not statistically significant.

The stability of the metabolomic profiles was initially assessed. As illustrated in the PCA plots (Figure S3), the serum QC samples in both ESI- and ESI+ modes clustered together. The serum metabolome composition was modeled using partial least squares-discriminant analysis, and biomarkers were identified based on their variable importance in projection (VIP) values. Several metabolites were identified as biomarkers with a VIP > 2.0 (VIP values of compounds were shown in Supplemental Table S4). Notably, changes in specific human serum metabolites were observed before and after CCFM1025 and placebo interventions. In the CCFM1025 intervention group, six biomarkers, including choline, gentian violet, oleamide, daidzein, pentadecanoic acid, and acetylcholine, were identified as key substances (Figure 5A). We first analyzed the relationship between these six biomarkers and three stress markers. The results revealed that only one substance, daidzein, exhibited a significant correlation with the stress markers (Figure 5C). Daidzein also served as a biomarker with a VIP > 2 in the placebo intervention group (Figure 5B). Therefore, we chose it for further analysis.

However, it is important to highlight that the relative abundance of daidzein in serum varied differently between two groups (Figure 5D). Although the content of daidzein increased in both groups after intervention, the relative abundance of daidzein in the serum of participants in the CCFM1025 group was significantly higher after intervention than before intervention (pre vs. post: t (19) = 2.29, *p* = 0.0337, d = 0.738; Figure 5D). In contrast, there was no significant difference before and after intervention in the placebo group (pre vs. post: t (19) = 0.1374, *p* = 0.8921, d = 0.044; Figure 5D).

Further investigation was conducted to examine the link between stress markers and the relative abundance of daidzein. The concentrations of stress markers, including salivary cortisol, plasma cortisol, and plasma ACTH, exhibited negative correlations with the relative abundance of serum daidzein, respectively (salivary cortisol vs. daidzein: R = −0.41, *p* < 0.001, Figure 5E; plasma cortisol vs. daidzein: R = −0.36, *p* < 0.001, Figure 5F; plasma ACTH vs. daidzein: R = −0.24, *p* < 0.05, Figure 5G).

## 4. Discussion

This study delved into the impact of *Bifidobacterium breve* CCFM1025 on sleep quality in individuals with insomnia. After a 4-week intervention, we observed a substantial improvement in sleep quality in the CCFM1025 group, as evidenced by a significant reduction in their PSQI scores in comparison to the placebo group. This improvement encompassed not only subjective sleep quality but also the alleviation of sleep disorders, particularly early waking at night, often associated with abnormal cortisol secretion (as illustrated in Figure 3A–C).

Stress has been found to be associated with the occurrence of insomnia episodes, being a major contributing factor among the various potential causes of the sleep disorder. When acute stressors are encountered, the production and release of corticotropin-releasing hormone (CRH) and arginine vasopressin (AVP) are increased, which in turn triggers the release of ACTH from the anterior pituitary. ACTH then stimulates the synthesis and release of cortisol from the adrenal cortex [28]. An overexcited and hyperactive HPA axis can lead to wakefulness, while regulating the HPA axis can be instrumental in enhancing sleep quality and, consequently, treating insomnia [29].

Insomnia’s onset and progression are closely linked to the dysfunction of the HPA axis [30]. Recent studies have highlighted the relationship between changes in HPA axis function and sleep disorders. For instance, individuals with poor sleep exhibit increased 24-h urine cortisol secretion [31], and there is a positive correlation between total waking time and 24-h urine cortisol secretion in individuals with mild insomnia [32]. Adolescents with insomnia show elevated plasma cortisol levels from 9 pm to 1 am [33]. These findings underscore the interplay between alterations in HPA axis function and sleep disturbances. In alignment with prior research on probiotics, our study did not uncover significant differences in the levels of stress-related hormones in saliva and blood before and after the intervention [12,34]. However, it is noteworthy that, in comparison to the placebo group, there was a more substantial reduction in participants’ salivary and plasma cortisol levels after the intervention with CCFM1025 (Figure 4A,B). This suggests that CCFM1025 may possess a regulatory effect on the HPA axis, and this effect might become more pronounced with extended intervention periods. This hypothesis is consistent with a recent meta-analysis, which demonstrated that participants receiving probiotic therapy for eight weeks or longer showed the greater improvements in mean PSQI score and sleep efficiency [35].

The intervention with CCFM1025 had an impact on the serum metabolites of participants. In our study, we identified a key substance, daidzein, which displayed differential expression in the serum before and after the experimental intervention in both the probiotic and placebo groups (Figure 5A,B). Daidzein, classified as a phytoestrogen isoflavonoid belonging to the category of nonsteroidal estrogens, boasts a diverse array of pharmacological effects [36]. Notably, daidzein has been implicated in the regulation of the endocrine system [37] and functions as an antioxidant [38]. Moreover, it exerts neuroprotective effects and exerts a beneficial impact on neurological disorders through multiple mechanisms., such as shielding nerve cells from death under hypoxic conditions by activating gamma receptors [39]. It enhances memory by upregulating the expression of brain-derived neurotrophic factor and stimulating the cholinergic system. It can also improve cognitive function through the activation of the p-CREB/BDNF pathway [40,41]. In primary cultured nerve cells, daidzein has demonstrated significant protection against beta-amyloid-induced neurotoxicity and glutamate excitotoxicity. Recent studies have also illuminated daidzein’s influence on the HPA axis. Chronic or sub-chronic daidzein administration has been found to alleviate elevated serum levels of stress-related hormones, namely, cortisol and ACTH, in both depressed and stressed model rats [42]. In our experiment, daidzein is an important serum biomarker. Following intervention with CCFM1025, there was a significant increase in the relative abundance of daidzein in serum (*p* < 0.05, Figure 5D), indicating that the administration of CCFM1025 can, to some extent, influence the levels of daidzein in the serum. Our correlation analysis further unveiled a negative relationship between stress indicators and the relative abundance of serum daidzein (Figure 5C–E), suggesting that changes in serum daidzein levels due to CCFM1025 intervention may influence HPA axis activity, enhance awakening behavior, and consequently improve sleep quality. However, the precise mechanisms underlying this process necessitate further exploration.

Our study has several limitations that warrant attention and should be addressed in future research endeavors. Firstly, it is essential to increase the number of participants in the second round of human trials, taking into account potential sex differences within the treatment group. This step will enhance the robustness and generalizability of our findings. Secondly, our study did not implement strict dietary controls for participants, with the exception of antibiotic use. Consequently, we were unable to determine whether diet directly influenced serum metabolite levels. Given that the gut microbiome is predominantly influenced by diet, it is possible that the specific impact of probiotics on the gut microbial profile remains unclear. To obtain more meaningful insights, future investigations should consider implementing rigorous dietary controls and employing shotgun metagenomics analysis. Undoubtedly, alterations in our living environment, work, social schedules, and biological elements can influence sleep to some extent [43]. Nevertheless, our study primarily concentrated on the connection between the independent application of probiotics and sleep quality, without addressing other potential influencing variables. In future research, these variables should be considered. Additionally, our study collected cortisol samples at a specific time, around 9:00 am, since it can represent the basal cortisol level [44]. To obtain a comprehensive understanding of serum cortisol levels, it is worthwhile to consider collecting samples at different times, as suggested in a recent study.

## 5. Conclusions

In line with the anticipated outcomes based on preclinical research, *Bifidobacterium breve* CCFM1025 emerges as a promising candidate among psychobiotic strains for enhancing sleep quality. The mechanisms underlying this effect may be linked to the influence of serum metabolites. Our study contributes fundamental insights into the potential use of psychobiotics to enhance sleep quality. However, to fully establish the therapeutic efficacy of CCFM1025 and delve deeper into the underlying mechanisms, further investigations are warranted. These future studies should encompass larger-scale clinical trials with varying durations of follow-up. Such endeavors will allow for a more comprehensive assessment of CCFM1025’s therapeutic potential and provide a detailed exploration of the mechanisms responsible for its beneficial effects on sleep quality.

## Figures and Tables

**Table 1:** Summary of Demographic Information.

Variable		Placebo (*n* = 20)	CCFM1025 (*n* = 20)	Statistic Value	*p* Value
Age, Mean ± SD		36.55 ± 11.31	38.95 ± 10.59	0.693	0.493 ^a^
BMI. Mean ± SD		22.59 ± 3.30	22.76 ± 3.05	0.168	0.866 ^a^
Sex, *n* (%)	Female	14 (70)	12 (60)	0.440	0.507 ^b^
	Male	6 (30)	8 (40)		
Race, *n* (%)	Chinese, Han nationality	20 (100)	20 (100)	N/A	N/A
Medication Before inclusion, *n* (%)		0 (0)	0 (0)	N/A	N/A
Educational status, n (%)	Primary	1 (5)	0 (0)	2.534	0.469 ^b^
	Secondary	3 (15)	4 (20)		
	Higher	10 (50)	13 (65)		
	Unknown	6 (30)	3 (15)		
Alcohol use, *n* (%)	Non-drinker	16 (80)	15 (75)	0.143	0.705 ^b^
	Occasional drinker	4 (20)	5 (25)		
Smokers, *n* (%)	Non-smoker	16 (80)	13 (65)	1.129	0.288 ^b^
	Smoker	4 (20)	7 (35)		

*n*, number of participants; N/A, not available; ^a^ Statistical analysis by *t*-test; ^b^ Statistical analysis by chi square test.

## Data Availability

The data presented in this study are available upon request from the corresponding author.

## Supplementary Materials

The following supporting information can be downloaded at: https://www.mdpi.com/article/10.3390/nu15214700/s1, Figure S1: Power analysis; Figure S2: Experimental schedule; Figure S3: Quality control assessment. Table S1: Demographic characteristics of all participants; Table S2: Statistical summary of PSQI and AIS; Table S3: Statistical summary of stress markers. Table S4: The VIP value of compounds.
