# Supplementary material for: Bifidobacterium breve CCFM1025 Improves Sleep Quality via Regulating the Activity of the HPA Axis: A Randomized Clinical Trial
Source: Nutrients. 2023 Nov 6;15(21):4700. doi: 10.3390/nu15214700 (PMC10648101; doi:10.3390/nu15214700)
Supplement: Supplementary file 1 [file nutrients-15-04700-s001.zip › nutrients-2697018-supplementary.pdf]

Supplementary Material

S1. Supplementary Methods

S1.1. UPLC-MS Analysis Parameters

Samples were analyzed using an ItiMateU-3000 ultraperformance liquid-chromatography (UPLC) system (Thermo Fisher Scientific, MA, USA) coupled to a high-resolution Q- Exactive mass spectrometer (Thermo Fisher Scientific, MA, USA). A Waters Acquity UPLC T3 column (2.1 × 100 mm, 1.8 μm) was used at an operating temperature of 30℃.

The mobile phases were A) 0.1% formic acid in negative mode or 5 mM ammonium acetate in negative mode and B) 100% acetonitrile. The analytical gradient was: 0–1 min, 5% B; 10 min, 99% B; 12 min, 99% B; 3.5 min, 95% B; 12.1 min, 5% B; 15 min, 5% B. The flow rate was 0.3 mL/min with an injection volume of 2 μL for both phases. Samples were held at 4℃ in the autosampler.

The Q Exactive was run with polarity switching (+3.80 kV/-3.20 kV) in full scan mode with an m/z range of 80–1050. The electrospray ionization (ESI) source conditions were set as follows: sheath gas flow of 40 psi, aus gas flow of 10 psi, capillary temperature of 320°C, and aus gas heater temperature of 350 °C. The normalized collision energy (NCE) was set to 20–40–60 eV.

S2. Supplementary Figures and Tables

S2.1. Supplementary Figures

TABLE 2. COMPARISON OF MEAN TOTAL PITTSBURGH SLEEP QUALITY INDEX SCORES AT 0, 2, AND 4 WEEKS OF INTERVENTION BETWEEN TWO GROUPS

Group	*Baseline*	*Second week*	*Fourth week*	*p-value within group*	*p-value between groups*
A (treatment), mean ± SD of total PSQI	11.7 ± 3.20	9.17 ± 3.68	7.55 ± 3.79	0.001	0.001
B (placebo), mean ± SD of total PSQI	12.09 ± 2.66	10.69 ± 3.42	10.66 ± 3.55	0.007	

PSQI, Pittsburgh Sleep Quality Index; SD, standard deviation.

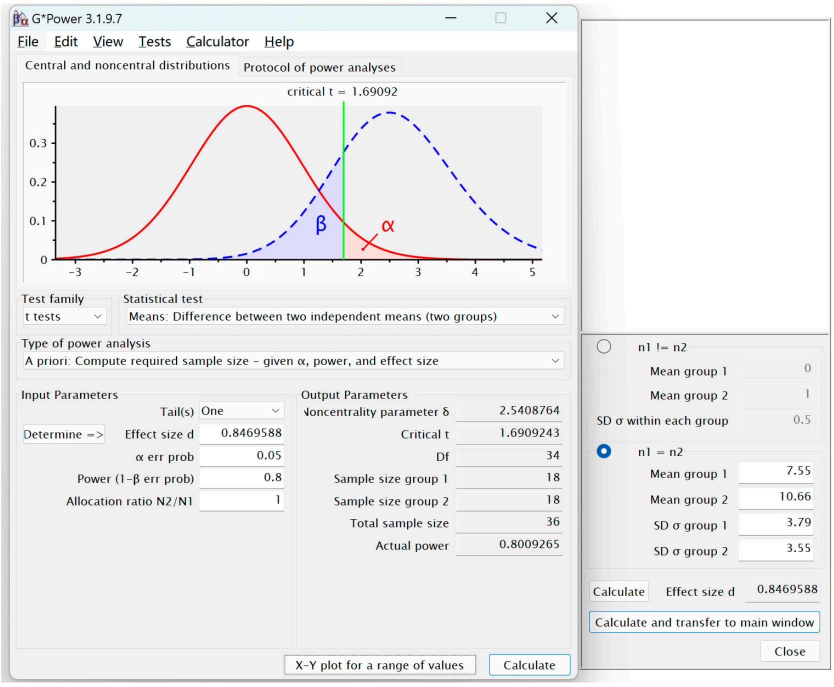

Figure S1. Power analysis. The estimated effect size was obtained from a published study that treat by comparing the mean difference of the Pittsburgh Sleep Quality Index score between the control and treated group.

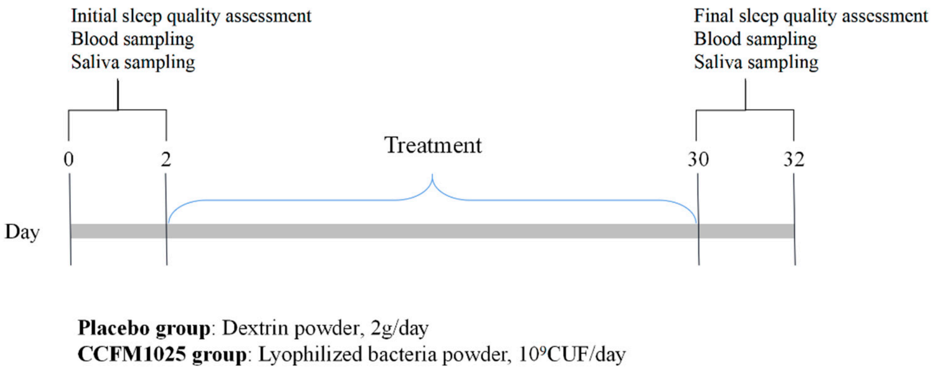

Figure S2. Experimental schedule.

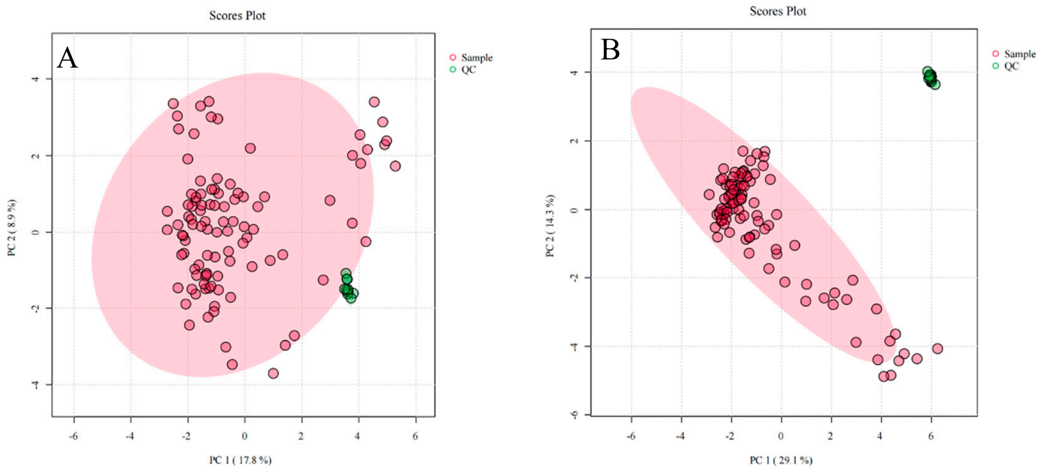

Figure S3. Quality control assessment (**A**) The PCA score plots for all serum samples containing QC samples in ESI+ mode. (**B**) The PCA score plots for all serum samples containing QC samples in ESI− mode.

S2.2. Supplementary Tables

Table S1. Demographic Characteristics of all participants.

Variable		Healthy Control (n = 20)	Sleep Disorders (n = 40)	Statistic Value	*p* value
**Age, Mean ± SD**		34.35 ± 8.93	37.75 ± 10.88	1.207	0.232 [a]
**BMI. Mean ± SD**		22.76 ± 2.85	22.67 ± 3.14	0.108	0.915 [a]
**Sex, n (%)**	**Female**	10 (50)	26 (65)	1.250	0.264 [b]
	**Male**	10 (50）	14 (35)		
**Race, n (%)**	**Chinese, Han nationality**	20 (100)	40 (100)	N/A	N/A
**Medication before inclusion, n (%)**		0 (0)	0 (0)	N/A	N/A
**Educational status, n (%)**	**Primary**	0 (0）	1 (2.5)	1.717	0.633 [b]
	**Secondary**	4 (20)	7 (17.5)		
	**Higher**	9 (45)	23 (57.5)		
	**Unknown**	7 (35)	9 (22.5)		
**Alcohol use, n (%)**	**Non-drinke**	17 (85)	31 (77.5)	0.469	0.494 [b]
	**Occasional drinker**	3 (15)	9 (22.5)		
**Smokers, n (%)**	**Non-smoker**	15 (75)	29 (72.5)	0.04	0.837 [b]
	**Smoker**	5 (25)	11 (27.5)		

n, number of participants; N/A, not available; [a] Statistical analysis by t-test; [b] Statistical analysis by chi square test.

Table S2. Statistical Summary of PSQI and AIS.

		M (SD)	F Value	*p* Value	Change	*p* Value
**CCFM 1025-PSQI**	pre	11.60 (3.169)	15.41	0.0007	3.850 (3.602)	0.0419
	post	7.75 (3.697)				
**Placebo-PSQI**	pre	10.10 (2.293)		0.4316	1.450 (3.605)	
	post	8.65 (2.793)				
**CCFM 1025-PSQI sleep quality**	pre	2.4 (0.598)	18.19	0.004	0.9 (0.9119)	0.0435
	post	1.5 (0.827)				
**Placebo-PSQI sleep quality**	pre	2.05 (0.394)		0.2557	0.35 (0.7452)	
	post	1.65 (0.813)				
**CCFM 1025-PSQI Sleep disturbance**	pre	1.65 (0.745)	0.937	0.038	0.6 (0.8826)	0.0032
	post	1.05 (0.605)				
**Placebo-PSQI Sleep disturbance**	pre	1.4 (0.681)		0.5226	-0.3 (0.9234)	
	post	1.7 (0.733)				
**CCFM 1025-AIS**	pre	11.5 (4.395)	13.46	0.0155	2.8 (6.058)	0.4601
	post	7.45 (2.282)				
**Placebo-AIS**	pre	12.75 (4.411)		0.1588	4.05 (4.407)	
	post	9.95 (5.073)				

Table S3. Statistical Summary of stress markers.

		M（SD）	F Value	P Value	Change	P Value
**CCFM 1025-salivary cortisol**	pre	16.72 (1.570)	1.67	0.0564	1.369 (1.118)	0.0019
	post	15.35 (1.322)				
**Placebo-salivary cortisol**	pre	15.74 (1.967)		0.8768	-5.222 (3.181)	
	post	16.14 (1.788)				
**CCFM 1025-plasma cortisol**	pre	17.94 (4.758)	0.088	0.5058	2.210 (4.593)	0.0312
	post	15.49 (5.748)				
**Placebo-plasma cortisol**	pre	16.26 (5.172)		0.7626	-1.716 (6.364)	
	post	17.97 (6.392)				
**CCFM 1025-plasma ACTH**	pre	51.03(26.48)	1.66	0.3141	13.56 (21.37)	0.0605
	post	37.47(18.40)				
**Placebo-plasma ACTH**	pre	39.69(22.39)		0.9997	0.6685 (20.78)	
	post	38.93(25.02)				

Table S4. The VIP value of compounds.

CCFM1025	Compounds	VIP	placebo	Compounds	VIP
	Choline	3.155359214		Hippuric acid	2.926772503
	Gentian violet	3.087027905		Gentian violet	2.641905497
	Oleamide	2.651032453		Sphingosine	2.586327598
	Daidzein	2.171505646		Valeric acid	2.477216246
	Pentadecanoic acid	2.162228648		Benzoic acid	2.472313739
	Acetylcholine	2.136958751		Pentaerythritol	2.378509372
	L-Citrulline	1.991798719		2-Amino-1,3,4-octadecanetriol	2.343947886
	Isobutyric acid	1.982251453		1-Methylxanthine	2.230131108
	Creatine	1.855916438		Daidzein	2.220437489
	Isoleucine	1.85155352		Eicosapentaenoic acid	2.05729217
	2-Naphthol	1.849716433		Glycochenodeoxycholic acid	2.056884017
	L-Threonine	1.823269132		Taurochenodeoxycholic Acid	1.959494064
	Dehydroacetic acid	1.808040047		Cyclamic acid	1.949377734
	2-Arachidonoyl glycerol	1.790125658		Ethyl violet	1.945619459
	DL-Glutamine	1.757140366		Hexanoic acid	1.922604942
	Piperine	1.748600042		Nonanoic acid	1.895399338
	Phytosphingosine	1.707309813		11-Deoxy prostaglandin F2	1.866562055
	Phloretin	1.684861605		4-Chlorophenoxyacetic acid	1.85721913
	Eicosenoic acid	1.664419617		Theophylline	1.854572555
	2-Furoic acid	1.646239615		Theobromine	1.828376463
